# Targeting Post-Translational Modifications of the p73 Protein: A Promising Therapeutic Strategy for Tumors

**DOI:** 10.3390/cancers13081916

**Published:** 2021-04-15

**Authors:** Ziad Omran, Mahmood H. Dalhat, Omeima Abdullah, Mohammed Kaleem, Salman Hosawi, Fahd A Al-Abbasi, Wei Wu, Hani Choudhry, Mahmoud Alhosin

**Affiliations:** 1 College of Pharmacy, Umm Al-Qura University, Makkah 21955, Saudi Arabia; zhomran@uqu.edu.sa (Z.O.); oaabdullah@uqu.edu.sa (O.A.); 2 King Fahd Medical Research Center, Cancer and Mutagenesis Unit, Department of Biochemistry, Faculty of Science, King Abdulaziz University, Jeddah 21589, Saudi Arabia; mdalhatdalhat@stu.kau.edu.sa (M.H.D.); mkaleem0002@stu.kau.edu.sa (M.K.); shosawi@kau.edu.sa (S.H.); abbasi@kau.edu.sa (F.A.A.-A.); hchoudhry@kau.edu.sa (H.C.); 3 Department of Medicine, University of California, San Francisco, CA 94143, USA; Wei.Wu@ucsf.edu

**Keywords:** p73, post-translational modification, ubiquitin, phosphorylation, acetylation, cancer therapy

## Abstract

**Simple Summary:**

The tumor suppressor TAp73 is a member of the p53 family, which is inhibited in many human solid and hematological tumors. In contrast to those in the *p53* gene, mutations in the *p73* gene are very rare in tumors, suggesting that the decrease in TAp73 activity and expression detected in those tumors are caused mainly by coordinated post-translational modifications of TAp73. Thus, understanding and investigating these post-translational modifications will allow for the identification of new targets to overcome the downregulation of TAp73 and, ultimately, the development of novel cancer therapeutics. This review highlights the multiple post-translational modifications underlying TAp73 regulation in cancer cells and the growing importance of targeting their trigger enzymes as a promising antitumor strategy.

**Abstract:**

The tumor suppressor p73 is a member of the p53 family and is expressed as different isoforms with opposing properties. The TAp73 isoforms act as tumor suppressors and have pro-apoptotic effects, whereas the ΔNp73 isoforms lack the N-terminus transactivation domain and behave as oncogenes. The TAp73 protein has a high degree of similarity with both p53 function and structure, and it induces the regulation of various genes involved in the cell cycle and apoptosis. Unlike those of the *p53* gene, the mutations in the *p73* gene are very rare in tumors. Cancer cells have developed several mechanisms to inhibit the activity and/or expression of *p73*, from the hypermethylation of its promoter to the modulation of the ratio between its pro- and anti-apoptotic isoforms. The p73 protein is also decorated by a panel of post-translational modifications, including phosphorylation, acetylation, ubiquitin proteasomal pathway modifications, and small ubiquitin-related modifier (SUMO)ylation, that regulate its transcriptional activity, subcellular localization, and stability. These modifications orchestrate the multiple anti-proliferative and pro-apoptotic functions of TAp73, thereby offering multiple promising candidates for targeted anti-cancer therapies. In this review, we summarize the current knowledge of the different pathways implicated in the regulation of TAp73 at the post-translational level. This review also highlights the growing importance of targeting the post-translational modifications of TAp73 as a promising antitumor strategy, regardless of p53 status.

## 1. Introduction

**Figure 1:**
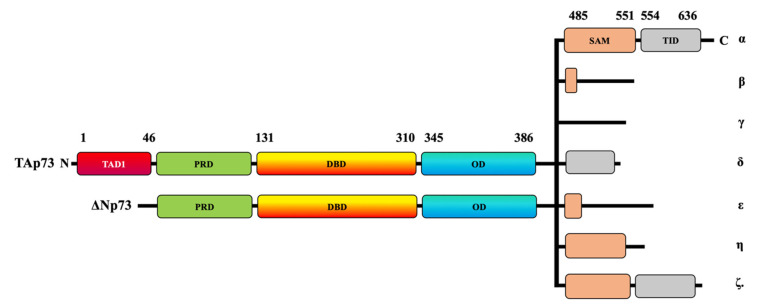
Schematic representation of TAp73 and ΔNp73 domains. The human p73 protein comprises transcriptional activating domains (TADs) and a proline-rich domain (PRD) at its N-terminal and a DNA-binding domain (DBD). The p73 C-terminal domain is composed of an oligomerization domain (OD), a sterile alpha motif (SAM) domain, and a transactivation inhibitory domain (TID). At least two N-terminal isoforms, TAp73 and ΔNp73, can be combined with the C-terminal α, β, γ, δ, ε, ζ, and η isoforms.

The *p53* gene, commonly referred to as the guardian of the human genome, is highly mutated in approximately 50% of human cancers [1,2]. It is a vital tumor-suppressor gene due to the ability of *p53* to induce apoptosis and growth arrest in response to cell stress, genotoxicity, and DNA damage [3]. Overall, p53 is a master transcription factor of cell cycle inhibitors, such as *p21^WAF1^* [4,5], as well as several pro-apoptotic genes, including *Bax* and *Bim* [6,7]. *p53* was thought to be a unique gene in the human genome for more than two decades until the discovery of two additional members of its family, *p63* and *p73*, which share a similar structure and function [8,9,10,11]. The tumor suppressor p53 and its homologs, p63 and p73, play crucial roles in the regulation of several cellular activities such as DNA damage response, development, cellular homeostasis, aging, and metabolism [12,13]. Like other members, p73 is also expressed as multiple functional isoforms (Figure 1). The *p73* gene has two different promoters, thereby allowing for the generation of two types of isoforms with two opposite functions. The transactivation-p73 (TA*p73*) isoforms contain the N-terminal transactivation domain with pro-apoptotic functions, whereas the N-terminally truncated p73 (ΔN*p73*) isoforms have anti-apoptotic properties (Figure 1) [11,14,15,16,17,18].

The human *p73* gene contains 14 exons that extend to around 80 kb. The p73 protein contains approximately eight domains. At its N-terminal, the human p73 protein comprises transcriptional activating domains (TADs), a proline-rich domain (PRD), and a DNA-binding domain (DBD). The carboxy-terminal (C-terminal) is composed of an oligomerization domain (OD), a sterile alpha motif (SAM) domain, and a transactivation-inhibitory domain (TID) [19,20]. The TID is a post-SAM region and is absent in p53 [20]. The TAp73 isoforms have multiple variants—α, β, γ, δ, ε, ζ, and η—that are expressed due to alternative splicing on the SAM domain located at the C-terminal region [21] (Figure 1). TAp73α is considered the longest variant because it has the longest SAM domain and the longest C-terminal, whereas p73β has a shorter SAM domain and a shorter C-terminal tail [20,22].

ΔNp73 lacks a transactivation domain (Figure 1), but it has oncogenic potential, as displayed by its dominant-negative effects on both wildtype p53 and TAp73 [16,17]. Like TAp73, ΔNp73 can be combined with C-terminal isoforms (Figure 1). ΔNp73 usually displaces wildtype p53 and TAp73 at the oligomerization level through competition for the DNA-binding site [23]. The overexpression of p73 induces apoptosis, whereas ΔNp73 can inhibit p73-induced cell death; therefore, the fine balance between the two variants can determine cell fate.

The increasing interest in the *p73* gene has resulted from the very low frequency of its mutations in human cancers [21,24,25]. Indeed, mutations in the *p73* gene are very rare in human tumors compared to those in the *p53* gene, which have been detected in more than 50% of human cancers [2,26,27]. In addition to *p73* gene mutations, *p73* has been also found to be epigenetically silenced in some leukemias and lymphomas through the hypermethylation of its promoter [28,29,30]. Alternatively, the loss of p73 in cancer may result from several post-translational modifications, including the ubiquitin-dependent proteasomal degradation pathway, phosphorylation, acetylation, and small ubiquitin-related modifier (SUMO)ylation. Thus, a deep understanding of p73 post-translational modifications will be extremely helpful in finding and developing new strategies for the prevention and treatment of cancers, especially those with p53 mutations. In this review, we summarize the current knowledge regarding the different pathways implicated in the regulation of p73 at the post-translational level. This review also highlights the growing importance of targeting the post-translational modifications of p73 as a promising antitumor strategy, regardless of the p53 status.

## 2. Ubiquitination-Dependent p73 Inhibition

In all cells, the intracellular proteins are degraded through the ubiquitin-proteasome pathway (UPP). The UPP contains specific enzymes that bind polypeptide chains to ubiquitin (Ub) and tag them for proteasomal degradation [31,32]. The tagged polypeptides are recognized by the 26S proteasome, a multicatalytic protease complex that degrades large ubiquitinated polypeptides into small peptides. Three enzymes are required for the ubiquitin tagging process: E1, a Ub-activating enzyme; E2, a Ub carrier or conjugating protein; and E3, a Ub protein ligase [33]. The E3 ligases are responsible for ligating Ub to polypeptides or proteins. By contrast, the deubiquitinating enzymes (DUBs) remove Ub from ubiquitinated proteins, thereby promoting protein stability.

**Figure 2:**
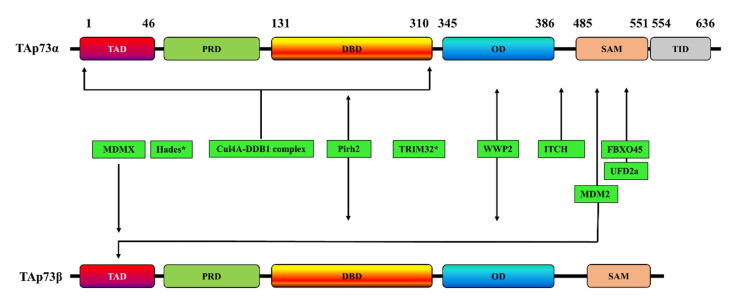
Schematic representation of the interaction sites of various E3 ubiquitin-ligases with TAp73α, TAp73β, or both. * indicates that the p73 interaction site is unknown. TAD: transactivation domain; PRD: proline rich domain; DBD: DNA binding domain; OD: oligomerization domain; SAM: sterile alpha motif; TID: transactivation inhibitory domain.

The reversible regulation of Ub by E3 ligases and DUBs has received increasing attention for several diseases, including cancers [34,35,36,37]. The ubiquitination pathway affects many tumor-suppressor proteins, including p53 and its homologs, p63 and p73 [38]. Over the last 20 years, researchers have discovered many ubiquitin E3 ligases that directly promote the protein degradation of p53, p63, and p73 by either ubiquitination-dependent or ubiquitination-independent proteasome pathways [39]. TAp73 was shown to undergo ubiquitination and to be inhibited by various E3 ubiquitin-ligase enzymes (Table 1) (Figure 2), such as Itch [40,41,42,43], mouse double minute 2 homolog (MDM2) and its homologue mouse double minute X (MDMX) [44,45,46], p53-induced RING-H2 protein (Pirh2) [47,48,49], tripartite motif 32 (TRIM32) [50], FBXO protein 45 (FBXO45) [51], Cullin4A (cul4A)-dependent ligase (CDL4A) [52], and WW Domain Containing E3 Ubiquitin Protein Ligase 2(WWP2) [53]. PIR2 (p73-induced ring protein 2) is also an E3 ubiquitin ligase for p73 through a mechanism that involves targeting the ΔNp73/TAp73α balance rather than the direct ubiquitination of p73 [54].

### 2.1. Itch

Itch E3 ubiquitin-protein ligase (Itch) has been reported to be highly expressed in several tumors, including lung cancer, breast cancer, pancreatic cancer, skin cancer, and neuroblastoma, and its inhibition leads to the induction of apoptosis and the inhibition of cell proliferation [38,59,62,63,64]. Itch selectively binds and ubiquitinates p73α via the PY motif (Met 452–Ala 489) but not p53, thereby leading to a proteasome-dependent p73 degradation [43]. The use of the proteasome inhibitor MG132 or the depletion of Itch using siRNA causes the inhibition of the Itch-mediated p73 degradation and increases p73 expression levels [43]. Similarly, treating cancer cells with DNA-damaging agents, such as doxorubicin, cisplatin, and etoposide, decreases the expression of endogenous Itch in a time- and dose-dependent manner, and this effect is associated with an increase in endogenous p73 levels and enhanced apoptosis [43].

Itch and p73 expressions are controlled through several mechanisms. For example, the Itch–p73 interaction was proposed to be under the control of the transcriptional co-activator Yes-associated protein 1 (Yap1), another partner of p73 [40]. Indeed, Yap1 competes with Itch for binding to p73, thereby preventing the Itch-mediated ubiquitination and degradation of p73. Cisplatin increases p73 expression and apoptosis, while the depletion of Yap1 using siRNA blocks these effects [40]. Yap1 interacts with another transcription activator, Runx, to target the promoter of Itch and enhance its transcription, with subsequent support of the degradation of p73 [41].Nedd4-binding partner-1 (N4BP1) has been also found to compete with Itch for binding to p73α, thus reducing the ability of Itch to interact with and ubiquitylate p73α [65]. The Itch/p73 axis is also regulated in chronic lymphocytic leukemia (CLL) cells through the direct regulation of Itch by microRNA 106b (miR106b) [42]. Indeed, the induction of miR106b in CLL cells is associated with a decrease in Itch expression and an increase in the expression of p73 and PUMA, as well as induction of CLL apoptosis [42].

### 2.2. MDM2/MDMX

MDM2 is a negative regulator of p53; its interaction with the TAD of p53 leads to a ubiquitination-mediated proteasomal degradation of p53 [13,66]. MDM2 also interacts with p73α and inhibits its function, but it does so without promoting p73 degradation [55,56]. MDM2 also forms a specific complex in vitro and in vivo with p73β through at least two residues (F15 and W19) [57]. MDM2 overexpression reduces the ability of p73β to activate transcription but without promoting its degradation, indicating that MDM2 can directly induce the degradation of the p53 protein but not of its homolog p73 [57]. However, MDM2 has been shown to induce p73 degradation in HeLa cells through a mechanism involving the interaction of MDM2 with Itch [45].

Adriamycin can increase p73 expression and HeLa cell apoptosis, most likely by decreasing the expression of both MDM2 and Itch. Similarly, the MDM2-mediated p73 degradation is inhibited in cells treated with the inhibitor proteasome MG132 [45]. In the same context, MDM2 was reported to use its RING domain to bind to p73α and p73β at multiple Lys residues of ubiquitin, thereby promoting the ubiquitination of both isoforms but without inducing their degradation [44]. MDM2 overexpression induces Itch-mediated p73 degradation in MDM2-deficient mouse embryo fibroblasts, whereas its depletion using siRNA induces p73-dependent transactivation and apoptosis [44].

A recent study showed that the MDM2/p73 interaction is established by specific binding between the N-terminus TA domain of MDM2 and the SAM domain of p73 [58]. MDMX, the structural homolog of MDM2, was also shown to negatively regulate p73 [46,59,67]. MDMX physically interacts with p73α, and its overexpression specifically induces alterations in the subcellular localization of p73α but not of the p73 homologs p53 and p63 [59]. Both MDM2 and MDMX are capable of binding to the TA domain of p73 [46].

### 2.3. Pirh2

Pirh2 is a RING finger E3 ligase that induces p53 degradation by promoting its ubiquitination [68,69]. Several studies have reported that Pirh2 acts as an oncogene, and its overexpression in cancer is associated with decreased p53 expression levels [48,49,70]. Pirh2 has also been shown to interact with the DNA-binding domain of both p73α and p73β [68,71,72], and its E3 ligase activity promotes p73 ubiquitination both in vivo and in vitro [47,73]. Pirh2 ubiquitinates p73 in vitro via K63-linked chains, while it promotes p73 ubiquitination in vivo by targeting K11-, K29-, K48-, and K63-linked chains [47]. The downregulation of Pirh2 significantly decreases p73 ubiquitination, whereas its overexpression is correlated with an inhibition of p73-dependent translational activity [47]. Pirh2 knockdown in the RKO colon carcinoma cell line inhibits cell growth and increases the levels of p73 and the cell cycle inhibitor p21^WAF1^ [73]. Conversely, the overexpression of Pirh2 in colon and breast cells induces the degradation of p73 via the proteasomal pathway [73]. The exposure of RKO cells to either doxorubicin or camptothecin decreases Pirh2-mediated p73 polyubiquitination [73].

### 2.4. TRIM32

Tripartite Motif Containing 32 (TRIM32) a RING protein, is overexpressed in several tumors, including skin tumors. It has E3 ubiquitin ligase proprieties and plays a role in tumor growth, metastasis, and resistance to anti-cancer drugs [74]. In this context, TRIM32 is involved in the ubiquitination of the tumor-suppressor Abl-interactor 2 (Abi2), resulting in an enhancement of cell proliferation and metastases [75]. TRIM32 also regulates the expression of p73 through the ubiquitination pathway, thereby promoting cell survival and tumor growth [50,76]. A regulatory feedback loop has been reported between TP73 and TRIM32 in human embryonic kidney 293 cells [50]. In HEK293 cells, p73 can act as a transcriptional regulator of TRIM32, and the depletion of p73 significantly decreases TRIM32 expression. TRIM32, in turn, binds to p73α and promotes its ubiquitination and degradation, thus inhibiting p73 transcriptional activity [50]. The overexpression of TRIM32 inhibits the p73 transcriptional activation of the p21^WAF1^ promoter, thereby enhancing cell cycle progression and cell survival [50].

### 2.5. FBXO45

Several studies have reported the overexpression of FBXO45 in human cancer and an essential role for FBXO45 in tumorigenesis and progression [77,78,79]. FBXO45 induces the ubiquitination–proteasomal degradation of several tumor-suppressor proteins, such as Par4 (prostate apoptosis response protein 4) [80], F-box/WD repeat-containing protein 7 (FBXW7) [81], and p73 [51]. Through its spla and the ryanodine receptor (SPRY) domain, FBXO45 specifically binds to the SAM domain of p73α, but not to its homolog p53, thus leading to the proteasome-dependent degradation of p73 both in vitro and in vivo [51]. The silencing of FBXO45 in the BT-20 cells, a p53-mutated cell line, induces the expression of p73 at the protein level [51]. The exposure of FBO45-depleted BT-20 cells to doxorubicin induces apoptosis, indicating that FBXO45 specifically binds to p73 and induces apoptosis via a p73-dependent mechanism [51]. Similar to p73, FBXO45 is also able to interact with ΔNp73 to play a crucial role in its stability in apoptosis and the DNA damage response. However, mutant FBXO45 has shown an impaired function against p73 and does not cause ubiquitination-associated p73 degradation [51].

### 2.6. UFD2a

Ubiquitin factor 2A (UFD2a), also known as Ubiquitin factor E4B (UBE4B), is an E3/E4 ligase belonging to the U box-type ubiquitin protein ligase family that can catalyze the elongation of the polyubiquitin chain. In response to apoptosis, UFD2a is cleaved by caspase 6, resulting in impaired enzymatic activity and dysfunction, as well as confirming the role of UFD2a in apoptotic signaling [82]. UFD2a physically interacts with p53 in brain tumors to promote p53 polyubiquitination and degradation, thus decreasing p53-mediated apoptotic activity [83]. Similar to its effects on p53, UFD2a regulates the stability of p73 via ubiquitination-independent degradation [60]. UFD2a also physically interacts with the SAM domain of p73α [60]. The treatment of SH-SY5Y neuroblastoma cells with cisplatin results in a decrease in the expression of UFD2a, while p73 protein expression and apoptosis are increased [60]. The expression of the p73 protein is also increased in UFD2a-depleted cells [60]. Alternatively, the overexpression of UFD2a decreases both p73α expression and p73-mediated pro-apoptotic activity [60]. Similar findings have been reported in squamous cell carcinoma (SCC) cells [84]. UFD2a knockdown in SCC cells was found to result in p73 accumulation and sensitized the SCC cells to the inhibitory effects of 1,25D3 (the most active metabolite of vitamin D) on cell growth, suggesting that the UFD2a/p73 pathway contributes to the antiproliferative effects of anticancer drugs [84,85].

### 2.7. Hades

Hades, also referred to as mitochondrial ubiquitin ligase activator of NFKB1 (MULAN), is an E3 ubiquitin ligase. Hades is anchored to the outer membrane of mitochondria through its transmembrane domains, with a conserved C-terminal RING-finger domain with an E3 ligase activity facing the cytosol [61]. Hades interacts with p53 in the mitochondria to reduce p53 stability [86]. Hades polyubiquitinates p53 through an MDM2-independent mechanism, thereby inhibiting the p53-dependent mitochondrial cell death pathway by blocking the interaction between p53 and Bcl-2 [86]. Through its RING-finger domain, Hades also interacts with p73 in etoposide-treated H1299 cells [61]. In response to etoposide treatment, p73 is translocated to the mitochondria to colocalize with Hades [61]. Hades induces the ubiquitination of p73 and its degradation, whereas its depletion using siRNA significantly inhibits p73 degradation, indicating that Hades is involved in the ubiquitination-dependent degradation of p73 [61].

### 2.8. The Cul4A–DDB1 E3 Ubiquitin Ligase Complex 

The CDL4A complex targets various proteins that are involved in several signaling pathways, including the DNA damage response and the cell cycle [87,88,89], and it contributes to the degradation of several proteins through the ubiquitin-proteasome pathway. In this context, Cul4A has been reported to interact with p53 to promote its proteasomal degradation [90]. Through damage specific DNA binding protein 1(DDB1), the CDL4A complex can directly bind to the N-terminal region of p73, which encompasses residues 1–320 and includes the DNA-binding domain, thereby inducing p73 mono-ubiquitination and inhibiting p73-dependent transcriptional activity without affecting p73 stability at the protein level [52]. The depletion of DDB1 induces the expression of several known target genes of p73, such as *p21^WAF1^* and *PUMA* [52]. Clinically, high expression levels of Cul4A in human breast carcinomas are associated with the repression of target genes of p73, indicating that Cul4A is a potent inhibitor of p73 transcriptional activity in cancer [52].

### 2.9. WWP2

WWP2, an E3 ubiquitin ligase, has an important role in different cellular functions and ubiquitinates, and it decreases the expression of the tumor-suppressor genes *PTEN* [91] and *p73* [53] through the proteasomal pathway. Through its WW3 domain, WWP2 specifically interacts with the oligomerization domain of both p73α and p73β but not with p53 [51]. This results in p73 ubiquitination via a K48 linkage, with the subsequent induction of p73 degradation in the proteasome [53]. Interestingly, WWP2 depletion dramatically decreases p73 ubiquitination and increases its expression at the protein level [53].

## 3. Phosphorylation-Dependent p73 Inhibition

**Figure 3:**
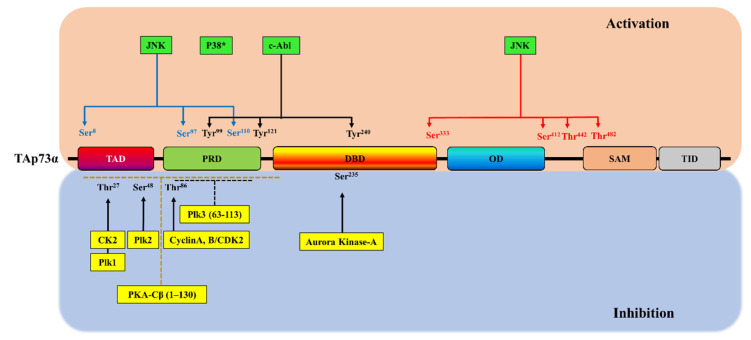
Positions of phosphorylation sites on TAp73α. Upper panel: phosphorylation-dependent activation of TAp73α and its phosphorylation sites. Lower panel: phosphorylation-dependent inhibition of TAp73α. * indicates that the p73 phosphorylation site is unknown.

The phosphorylation of p73 through its interaction with several kinases is considered an essential pathway for the regulation of p73 activity and stability under normal conditions and in response to DNA damage (Figure 3, upper panel). Several studies have reported that p73 is phosphorylated at Tyr^99^ by the non-receptor tyrosine kinase c-Abl in response to DNA damage, resulting in increases in p73 stability and pro-apoptotic activity [92,93,94]. In addition, p73 is phosphorylated and activated in response to genotoxic stress through its association with other kinases such as the protein kinase Cδ catalytic fragment at Ser^289^ [95]. In addition to this phosphorylation-dependent activation of p73, several reports have shown that various kinases can trigger the phosphorylation of p73 in response to DNA damage agents, leading to the inhibition of the p73 pro-apoptotic activity (Table 2; Figure 3, lower panel).

### 3.1. Cyclin-Dependent Kinase Complexes 

Physical interactions occur between p73 and several cyclins (A, B, D, and E) [96]. Several p73 isoforms, notably α, β, and γ, are phosphorylated at threonine 86 (Thr^86^) by cyclin-dependent kinase (CDK) complexes, including cyclin A/CDK2 and cyclin B/CDK2, in a cell cycle-dependent manner both in vitro and in vivo [96]. The phosphorylation of p73 is induced during the S phase and reaches maximal levels in the G2/M phase. CDK complexes induce p73 phosphorylation at Thr^86^ to decrease p73 transcriptional activity on the expression of the *p21^WAF1^* gene, but this effect is inhibited when Thr^86^ is mutated [96].

### 3.2. Protein Kinase A

A study using a yeast two-hybrid system has shown that the protein kinase A catalytic subunit (PKA-C) β is a novel partner of p73 [97]. Unlike p53, p73α is associated with PKA-Cβ, and this interaction has been found to occur in the nucleus of p53-deficient H1299 cells [97]. PKA-Cβ interacts with both the C-terminal (469–636) and the N-terminal (63–130) regions of p73α, and it bridges these regions to render p73α in an inactive form [97]. Through its kinase activity, PKA-Cβ phosphorylates p73α at its N-terminal region (residues 1–130), which leads to the inhibition of the p73 transcriptional activity and the suppression of the expression of the *p21^WAF1^* and *Bax* genes [97]. A kinase-deficient mutant of PKA-Cβ (K76R) was found to not be able to induce these inhibitory effects on p73 activity [97].

### 3.3. Polo-Like Kinase Family Members

Through its NH_2_-terminal region, p73α can interact with the amino acid 99–218 region of Polo-like kinase1 (Plk1), which includes a kinase activity [98]. Plk1 phosphorylates p73α at Thr^27^, resulting in the inhibition of its pro-apoptotic activity [98]. The Plk1-induced phosphorylation of p73α at Thr^27^ decreases the p73 transcriptional activity for the expression of the *p21^WAF1^*, *Bax,* and *MDM* genes, as well as p73 pro-apoptotic activity [98]. The treatment of COS7 cells (a p53-deficient cell line) with cisplatin was found to induce apoptosis, and this effect has been associated with a decrease in the expression of Plk1 and an increase in the expression of p73α, thereby indicating a negative regulation of the activity and stability of p73α by Plk1. The same findings have been observed after the downregulation of Plk1 in p53-deficient H1299 cells. The depletion of Plk1 in H1299 was found to further result in apoptosis that was correlated with an increase in p73α expression [98].

The Polo-like kinase family member Plk3 also interacts with p73α at its NH_2_-terminal region (the amino acids 63–113) and phosphorylates p73α in this portion, thereby inhibiting its pro-apoptotic activity [99]. Like Plk1 [98], the kinase activity of Plk3 can phosphorylate p73α (mostly at amino acid residues 63–113), leading to a decrease in transcriptional p73α activity on the expression of *p21^WAF1^* and the pro-apoptotic genes *Bax* and *p53AIP1*. The knockdown of Plk1 in H1299 increases the expression of the p73α protein, indicating that Plk3 exerts an inhibitory action against p73α activity and stability [99].

In p53-null Saos2 osteosarcoma cells, Plk2 can physically bind to and phosphorylate p73 at Ser^48^, located in the p73 TA domain, when p73 is upregulated in response to cisplatin or Adriamycin [100]. This p73 phosphorylation is not observed when Ser^48^ is mutated. The incubation of cisplatin-treated Saos2 osteosarcoma cells with a PLK2 inhibitor or PLK2 depletion using siRNA increases the expression of the *p21^WAF1^* and *PUMA* genes, inhibits cell proliferation and cell invasion, arrests the cell cycle at the G1 phase, and induces apoptosis, thus indicating that PLK2 phosphorylates p73 and inhibits p73 transcriptional activity [100].

### 3.4. Aurora Kinase-A

Aurora kinase-A is overexpressed in a panel of human tumors [103,104,105] and serves as a novel partner and negative regulator of p73 through phosphorylation [101]. Aurora kinase-A can phosphorylate p53 at the Ser^315^ [106] and Ser^215^ residues [107]. Aurora kinase-A can also induce p73 phosphorylation at Ser^235^, a residue located in the p73 DNA-binding domain. This decreases the ability of p73 to bind to DNA, thereby impairing its transactivation activity and, by consequence, its ability to inhibit apoptosis [101].

### 3.5. CK2

Casein kinase II (CK2) expression is dysregulated in many tumors [108,109,110] and can negatively regulate the expression of p53 in head and neck squamous cell carcinoma (HNSCC) [111]. CK2 also interacts with and phosphorylates p73 at the threonine 27 residue (T^27^), located in the N-terminal domain of p73, resulting in the inhibition of p73 activity [102]. This CK2-induced inhibition of p73 activity has been associated with the overexpression of several cancer stem cell markers, including Nanog, Oct4, and Sox2 [102]. Interestingly, CK2 inhibition or its knockdown can increase the expression levels of the p73 mRNA and protein and decrease the expression of Nanog, Oct4, and Sox2 [102].

## 4. Deacetylation-Dependent p73 Inhibition

**Figure 4:**
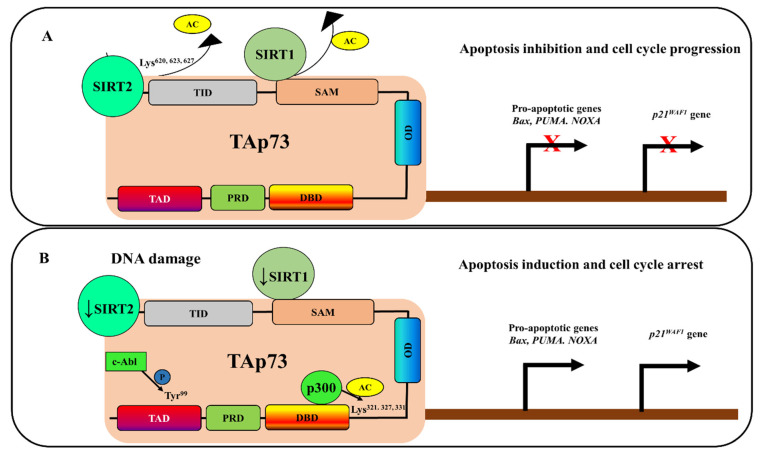
Schematic model of the role of the acetylation pathway in TAp73 regulation. (**A**) SIRT2 can bind to p73α and suppress its transcriptional activity through the deacetylation of several lysine residues located in the p73α C-terminal region: Lys^620,623,627^. SIRT1 can also bind to and deacetylate the p73 protein to inhibit apoptosis and promote cell cycle progression through the inhibition of the pro-apoptotic genes *Bax*, *PUMA,* and *NOXA* and the cell cycle inhibitor *p21^WAF1^* gene. (**B**) In response to DNA damage, the expression of SIRT1 and SIRT1 proteins is downregulated, while the acetyltransferase p300 is activated and its binding to TAp73 is promoted. TAp73 is then acetylated at several lysine^321,327,331^ positions. The p300-mediated TAp73 acetylation is boosted by its phosphorylation at tyrosine^99^ by c-Abl, thereby promoting p73 activity and stability. The end result is an induction of apoptosis through the reactivation of *Bax, PUMA,* and *NOXA* and the arrest of the cell cycle via the activation of *p21^WAF1^*.

Like p53, which is inactivated through Sirtuin (SIRT)1-mediated deacetylation at lysine 382 [112,113], p73 transactivation is also negatively regulated through a deacetylation process in human tumors [114,115]. In this context, SIRT1 has been shown to bind and deacetylate the p73 protein, thereby inhibiting the p73-dependent transcriptional activity on the expression of the *Bax* gene (Figure 4A) [114]. The overexpression of SIRT1 and p73 in HeLa cells inhibits ionizing radiation-induced apoptosis, but the downregulation of SIRT1 promotes apoptosis via a p73-dependent mechanism [114]. The p73 activity was also inhibited through acetylation by SIRT2 in glioblastoma cells [115]. The acetylation of p73 by SIRT2 confirmed the requirement for SIRT2 for the growth and survival of human glioblastoma cells, as well as for the formation and progression of glioblastomas in mouse models [115]. The deacetylase activity of SIRT2 suppresses the transcriptional activity of p73α through the deacetylation of several lysine residues (Lys^620^, Lys^623^, and Lys^627^) located in the C-terminal region of p73α (Figure 4A) [115]. In p53-mutated GB2 cells, the knockdown of SIRT2 or its inhibition using a specific inhibitor, AGK2, decreased cell proliferation and increased the expression of cleaved caspase 3 and the pro-apoptotic genes *PUMA* and *NOXA*. Conversely, the growth suppression induced by SIRT2 knockdown was rescued when p73 was downregulated in p53-mutated GB2. Mechanistically, the SIRT2 and p73 proteins were co-localized in the nucleus in glioblastoma patient samples, suggesting that the SIRT2-mediated deacetylation of p73 is a major cause of the inhibition of p73 transcriptional activity that allows glioblastoma cells to escape p73-mediated proliferation arrest and apoptosis [115].

Similar to p53, p73 is also regulated through acetylation mediated by the histone acetyltransferase p300. Through its NH_2_-terminal transactivation domain, p73 binds to the NH_2_-terminal CH1 domain of p300 and stimulates its p73-mediated transcriptional activation, as well as apoptosis [116]. A subsequent study reported that four domains of p300 can bind to p73 and affect its transcriptional activity [117]. In response to DNA damage, p73 is acetylated by p300 at several lysine positions (Lys^321^, Lys^327^, and Lys^331^) [118], and this p300-mediated acetylation of p73 first requires p73 phosphorylation by c-abl (Figure 4B) [119]. This indicates that the activation of p73 may require a coordinated collaboration between several post-translational modifications, such as the phosphorylation of its N-terminus by c-abl to increase the affinity of this p73 domain for acetylation by p300 in order to increase p73 transactivation (Figure 4B). This possibility is in line with the early statement showing that p300-mediated p73 acetylation is regulated in a c-abl-dependent manner through p73 phosphorylation at a tyrosine (Y99), which then promotes p73 activity and stability, as well as triggering the arrest of the cell cycle at the G1/S stage through the activation of p21^WAF1^ [119].

## 5. SUMOylation-Dependent p73 Inhibition

The SUMOylation of proteins is catalyzed by SUMO and this process is considered a key post-translational modification that regulates the expression of several targets in cancers, including tumor suppressor p53 [120,121,122,123]. Like p53, p73 is also post-translationally modified by SUMOylation [124,125]. The protein inhibitor of activated signal transducer and activator of transcription (STAT)-1 (PIAS-1), a SUMO E3 ligase [126,127], can bind to a region (aa 345–450) that contains the OD of the p73 isoforms α, β, and γ [124]. Through the RING finger domain, PIAS-1 is able to SUMOylate p73α, and this modification occurs in the nucleus matrix and decreases the p73 transcriptional activity on several genes, such as *Bax* and *MDM* [124]. PIAS-1 is mainly expressed during the S phase, and its overexpression decreases the p73-dependent transcription of the *p21*^WAF1^ gene, leading to a decrease in the numbers of cells in the G1 phase and supporting a role for PIAS-1 mediated-p73 SUMOylation in the regulation of cell cycle progression [124].

PIASγ, another member of the PIAS SUMO-ligase family, mimics the effect of PIAS-1 on p73α [125]. PIASγ can bind and SUMOylate p73a, thereby promoting p73a proteasomal degradation. PIASγ overexpression in HEK293 cells inhibited the p73a-mediated transcription of *p21*^WAF1^, and this effect was associated with a G1-to-S phase transition, providing further evidence for an important role of PIAS-mediated p73 SUMOylation in the regulation of the cell cycle [125].

## 6. Targeting Post-Translational Modifications of p73 for Cancer Therapy

Drug resistance is one of the major challenges in the field of cancer therapy [128,129]. In cancers with p53 mutations, which are detected in more than 50% of human cancers [2,26,27], the inhibition of the p73 transcriptional activity on the expression of cell cycle inhibitors, including *p21*^WAF1^, and pro-apoptotic genes, such as *Bax* and *PUMA,* is an important mechanism by which cancer cells escape apoptosis and develop resistance to chemotherapy. Cancer cells have developed several mechanisms to inhibit p73 expression, ranging from the hypermethylation of the p73 promoter in some leukemias and lymphomas [28,29] to the modulation of the ratio between the pro- and anti-apoptotic p73 isoforms in various other tumors [17,118,130].

**Figure 5:**
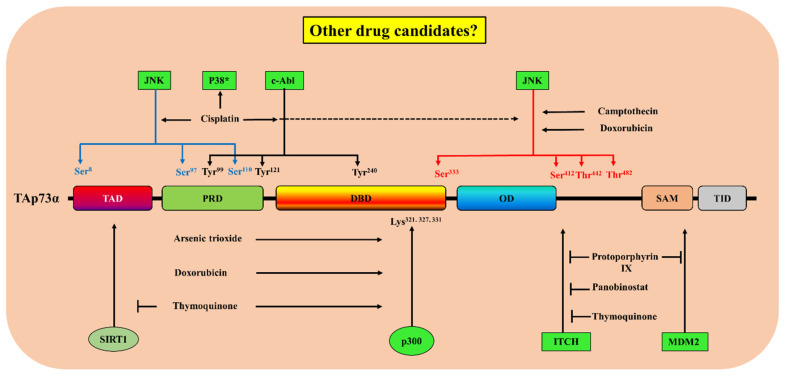
Summary of the effects of drugs targeting post-translational modifications of TAp73. These compounds are either activators or inhibitors of one or several pathways involved in the regulation of TAp73 at the post-translational level. A question mark emphasizes the possibility that other drug candidates can target post-translational modifications of TAp73 for cancer therapy.

As summarized above, p73 is also decorated by a panel of post-translational modifications that regulate its transcriptional activity, subcellular localization, and stability. These modifications orchestrate the multiple anti-proliferative and pro-apoptotic functions of TA p73, thereby offering various promising candidates for targeted anti-cancer therapies. In cells lacking functional p53, finding anticancer agents that can induce apoptosis by targeting the post-translational modifications of p73 has attracted substantial interest. Several compounds including Food and drug administration (FDA)-approved drugs have been identified as regulators of post-translational modifications of p73 to restore TAp73 transcriptional activity (Table 3 and Figure 5). The administration of some of those drugs such as cisplatin [131], Panobinostat [132], and doxorubicin [133] among cancer patients is highly controlled because it is associated with various side effects. Since the expression and/or the activity of TAp73 is inhibited in several human tumors through several mechanisms, the use of a molecular prescreening program in cancer patients to identify the mechanism of p73 dysfunction is needed to adopt an effective therapy.

### 6.1. Targeting the p73 Ubiquitination Pathway 

Protoporphyrin IX (PpIX), a natural metabolite of δ-aminolevulinic acid, is an anticancer drug used in clinics as a cream under the name “Metvix^®^” for the treatment of skin cancer [143,144]. Several tumor suppressors, including p53 [134,145] and p73 [67,135,146], have been reported as PpIX targets. PpIX can directly bind to the N-terminal domain of p53, thereby disrupting both the p53/MDM2 and p53/MDMX complexes, with the consequence of inducing apoptosis [134]. PpIX can also bind to the N-terminal domain of p73 to activate and stabilize this for the later induction of p73-dependent apoptosis in p53-deficient colon cancer cells [135]. The p73-dependent apoptosis in response to PpIX is associated with the upregulation of *NOXA* and *PUMA*, the pro-apoptotic targets of p73. The depletion of p73 results in cell resistance to PpIX and abolishes the PpIX-induced upregulation of both *NOXA* and *PUMA* [67].

PpIX can also inhibit the growth of HCT116 (p53nul) xenograft tumors in mice, and this effect is associated with an increase in the expression of the p73 protein. Mechanistically, PpIX can activate and stabilize the p73 protein in p53-mutated colon cancer cell lines (H1299 and HCT 116) through the disruption of both the p73/MDM2 and p73/MDMX complexes [67]. In effect, this mimics the effects on p53 activation in wildtype p53 cells [134]. The PpIX-induced p73 stabilization also involves the inhibition of its interaction with the Itch E3 ligase [67]. Taken together, these findings indicate that PpIX can activate p73 in tumors by inducing its release from the MDM2 and MDMX proteins. This leads to p73 stabilization through the disruption of its interaction with Itch, a key E3 ubiquitin ligase responsible for ubiquitination-dependent p73 proteasomal degradation.

The drug panobinostat, a pan-deacetylase inhibitor, is clinically approved by FDA for the treatment of multiple myeloma under the name Farydac^®^ (LBH589) [147,148] and is currently under clinical investigation for the treatment of several solid tumors and hematological cancers worldwide [149,150]. In general, p73 appears to be a main target of panobinostat for the induction of p53-independent apoptosis in CLL cells [42]. The exposure of primary CLL cells to panobinostat was found to significantly increase the expression of the p73 protein and its downstream target PUMA, with a subsequent induction of apoptosis without affecting the levels of p53 [42]. In response to panobinostat, the expression levels of p73 protein were increased in 16 of 20 CLL samples, indicating that the panobinostat-triggered upregulation of p73 predominantly occurs due to post-translational mechanisms rather than the transcriptional induction of p73 mRNA. Interestingly, p73 upregulation in leukemia samples is correlated with a significant decrease in the levels of the Itch protein, suggesting that panobinostat increases p73 stabilization through the inhibition of Itch-mediated proteasomal degradation. In line with this hypothesis, the knockdown of Itch in K562 leukemia cells was found to be accompanied with an increase in p73 levels. Moreover, the panobinostat-triggered downregulation of Itch in CLL cells was attributed to E2F1 and myc-regulated transcription induction in the levels of miR106b [42]. These findings revealed that post-translational modifications of p73 have key roles in the induction of apoptosis induced by panobinostat in CLL cells. This has important therapeutic implications for this drug in CLL, as well as in other tumors with p53 mutations.

We and others have shown that thymoquinone (TQ), the bioactive compound of black seed oil, has inhibitory effects on several cancers through the targeting of several signaling pathways [151,152,153,154,155,156,157,158,159], including the reactivation of the p73 protein in p53-mutated tumors [136,160,161]. Recently, TQ was proposed to increase the expression of the p73 protein in a p53-mutant acute lymphoblastic leukemia cell line (Jurkat cells) by targeting the expression of various E3 ubiquitin ligases for p73, including Itch, Mdm2, FBXO45, and TRIM32 [62]. TQ increased the expression of the p73 protein in Jurkat cells, and this effect was associated with a significant decrease in the expression of Itch in those cells, as well as in human promyelocytic leukemia HL60 cells and human triple-negative MDA-MB-468 breast cancer cells that also bear mutant p53 [62].

### 6.2. Targeting of the p73 Phosphorylation Pathway

Following DNA damage, the p73 phosphorylation occurring through its interaction with kinases is also essential for the induction of its activity and stability. The c-Abl kinase is considered the main protein involved in the phosphorylation of p73. This phosphorylation subsequently leads to p73 accumulation and the induction of apoptosis in response to several DNA-damaging agents, including two approved drugs, cisplatin [137,140] and doxorubicin [119]. The treatment of HCT116 cells with cisplatin increases p73 protein expression and induces apoptosis, effects attributed to the activation of c-Abl [140]. The c-Abl-mediated p73 stabilization involves the binding of the SH2 domain of c-Abl to the p73 protein and results in p73 phosphorylation, mainly on Tyr-99, Tyr-121, and Tyr-240 [137]. The c-Abl-mediated phosphorylation of p73 in response to cisplatin appears to involve the activation of p38 and blocking this p38 activation prevents the subsequent c-Abl-induced accumulation of p73 [138]. This indicates that the activation of kinase pathways is required for the p73-mediated apoptosis induced by DNA-damaging agents such as cisplatin. In support of this idea, p73 was reported to be a substrate of the c-Jun N-terminal kinase (JNK) and that the activation of JNK by cisplatin is essential for p73-mediated apoptosis in p53-mutated lung cancer cells [139].

JNK forms a complex with p73α and phosphorylates it at several serine/threonine residues, located within both the N- and C-terminal fragments of p73α [139]. Therefore, while cisplatin increases the levels of the p73α protein, the inhibition of JNK using the inhibitor SP600125 or JNK depletion by siRNA inhibits the cisplatin-induced p73α upregulation [139]. Moreover, the exposure of wildtype mouse embryonic fibroblast cells to different DNA-damaging agents (cisplatin, doxorubicin, or camptothecin) increased the expression of the p73α protein, but no similar effect was observed in JNK-deficient cells [139]. These findings indicated that protein kinases, including c-Abl, p38, and JNK, play critical roles in the apoptosis induced by DNA-damaging agents by promoting p73 phosphorylation and increasing p73 stability and pro-apoptotic activity. This suggests that the modulation of protein kinase activity could be a promising strategy for p73 stabilization in many cancers, especially those that lack a functional p53 but express p73.

A leaf extract (PBL) from *Piper betel*, a medicinal plant from the Piperaceae used in chemoprevention for several cancers [162,163], has been shown to induce apoptosis and cell cycle arrest in hepatocellular carcinoma Hep3B (p53 null) cells by increasing the expression of p73, phosphorylated-p73, p21, Bax, and caspase-3 [164]. The phosphorylation of p73 induced by PBL is associated with the activation of several kinases, such as ataxia telangiectasia mutated (ATM), c-Abl, JNK, and p38 [164]. Interestingly, the administration of PBLs in a Hep3B-bearing xenograft was found to inhibit tumor growth while significantly increasing the expression of p73 and phosphorylated-p73 and the activation of JNK and p38. These findings provided additional evidence that targeting the phosphorylation of p73 could be a new promising strategy for cancer therapy [164].

### 6.3. Targeting of the p73 Acetylation Pathway

Like p53, p73 can be acetylated by the p300 histone acetyltransferase on several lysine residues. Notably, this acetylation is an essential post-translational modification for the regulation of p73 activity and stability [117]. Several studies have shown that p73 directly interacts with p300 and its homolog, CREB-binding protein (CBP) [116,117,165]. The treatment of HCT116 cells by doxorubicin induced the accumulation and acetylation of p73α by p300, predominately at lysine residues 321, 327, and 331 [119]. The p300-induced acetylation of p73α activated its pro-apoptotic activity, as reflected by the induction of the expression of *p53AIP1* in response to doxorubicin. Interestingly, the phosphorylation of p73α by the tyrosine kinase c-Abl appears to be an indispensable event for p73α acetylation by p300 in response to DNA damage [119]. For example, p73 can bind via its N-terminal domain to four domains of p300, and this interaction is required for p73 transcriptional activity on the *Bax* gene [117].

The c-Abl-mediated phosphorylation of p73 in response to DNA damage requires the prolyl isomerase Pin1, which promotes a conformational change in p73 and a subsequent enhancement of acetylation of p73 by p300 [166]. In the same context, the p300-mediated acetylation of p73 is promoted by the binding of YAP1 to the p73/p300 complex and contributes to the stability of p73 while enhancing p73-dependent apoptosis in response to DNA damaging agents, including cisplatin and doxorubicin [141]. Therefore, the acetylation of p73 by p300 is likely stimulated by several mechanisms that involve the negative regulation of p73 by SIRT1 and the positive regulation of p73 through its phosphorylation by c-Abl, JNK, p38, and ATM. This supports the idea that acetylation of p73, regardless of how it is induced, is likely to play a critical role in the ability of p73 to act as a tumor suppressor in cancers lacking functional p53.

This hypothesis is supported by reports showing that the exposure of the NB4 and K562 leukemia cell lines that lack active p53 to either arsenic trioxide (ATO) or MEK1 inhibitors (PD98059 and PD184352) increases the expression of the p73α protein, decreases ΔNp73, and induces apoptosis [142]. The combination of ATO and MEK1 inhibitors was found to synergistically increase the expression of the p73α protein, as well as its acetylation by p300 [142]. Interestingly, at apoptotic concentrations, ATO was found to induce the acetylation of p73α by increasing its interaction with p300, and this effect was also associated with a slight increase in p73α tyrosine phosphorylation in both the NB4 and K562 cell lines [142]. Moreover, the p300-mediated acetylation of p73α and its phosphorylation in the ATO-treated cells increased the recruitment of p73α to the promoters of the pro-apoptotic genes *Bax* and *p53AIP1* [142].

Recently, TQ was shown to increase the expression of the p73 protein and apoptosis in p53-mutated Jurkat cells in a dose- and time-dependent manner [136]. The TQ-induced upregulation of p73 was correlated with a sharp decrease in the expression of the SIRT1 protein, as well as an increase in the expression of p300 in Jurkat and breast cancer cells [136]. These observations indicate that TQ induces the reactivation of p73 via its acetylation through a mechanism involving the downregulation of the SIRT1 deacetylase and the upregulation of the p300 acetyltransferase, leading to apoptosis [136]. This mechanism could also involve the activation of the phosphorylation process to promote the acetylation of p73 and increase its stability. This hypothesis is supported by several reports that have shown that TQ can induce the activation of JNK and/or p38 MAPKs to trigger cell death and suppress metastasis in colon cancer cells [167,168,169]. Taken together, these findings indicate that p73 may undergo coordinated post-translational modifications as early events, including its phosphorylation and acetylation through direct interactions with kinases and the p300 acetyltransferase, in response to anticancer drugs such as ATO and TQ. This can allow p73 to function as a tumor suppressor in malignancies, regardless of the p53 status (Figure 4).

## 7. Conclusions

The downregulation of the tumor suppressor TAp73 represses the transcription of several anti-proliferative and pro-apoptotic genes in many human solid and hematological tumors. This, in turn, inhibits apoptosis and enhances cell proliferation. Via its structural domains, which show a high degree of similarity with those of p53, TAp73 interacts with several E3 ubiquitin ligases, kinases, and deacetylases. These proteins, which work together to orchestrate the multiple anti-proliferative and pro-apoptotic functions of TAp73, also offer multiple promising candidates for cancer therapy. Since mutations in the *p73* gene are very rare in tumors, unlike the case for the *p53* gene, the decrease in both activity and quantity of TAp73 detected in various human tumors could arise, in large part, from coordinated post-translational modifications of TAp73. This review has highlighted the multiple post-translational modifications underlying TAp73 regulation in cancer cells and the growing importance of targeting their trigger enzymes as a promising antitumor strategy. Thus, understanding the post-translational modifications involved in TAp73 regulation will allow for the identification of new targets and the exploration of new drugs that can increase the expression of TAp73. This will allow cancer cells to undergo apoptosis, regardless of their p53 status, through the reactivation of several pro-apoptotic genes.

## Figures and Tables

**Table 1:** Role of E3 ligases in p73 stability. TRIM32: tripartite motif 32; Cul4A: Cullin4A; MDM2: mouse double minute 2 homolog; Pirh2: p53-induced RING-H2 protein.

E3 Ligase	TAp73 Isoform	p73 Interaction Site	Effects on p73	Refs
ITCH	p73α	PY motif (Met 452–Ala 489)	Proteasome-dependent p73 degradation	[43]
MDM2	p73α	-	p73α activity inhibition but without promoting its degradation	[55,56]
	p73β	Two residues (F15 and W19)	p73β activity inhibition but without promoting its degradation	[57]
	p73α	-	p73 degradation through a mechanism involving the interaction of MDM2 with Itch	[45]
	p73α and p73β	N-terminal domain	p73 ubiquitination but without promoting its degradation	[44]
	p73α	The C-terminal SAM domain (especially the peptide comprising α4 and α5 helices of SAM)	-	[58]
MDMX	p73α	TA domain	Subcellular localization of p73 and inactivation of transcription	[46,59]
Pirh2	p73α and p73β	DNA-binding domain	p73 ubiquitination and repressing p73-dependent transcriptional activity	[47]
TRIM32	p73α	-	p73 ubiquitination and degradation, inhibiting p73 transcriptional activity	[50]
FBXO45	p73α	SAM domain	Proteasome-dependent degradation of p73	[51]
UFD2a	p73α	SAM domain	Ubiquitination-independent p73 degradation	[60]
Hades	-	-	Ubiquitination of p73, promoting its degradation	[61]
Cul4A–DDB1 complex	p73α	The N-terminal region of p73, encompassing residues 1–320 and including the DNA-binding domain.	p73 monoubiquitination and inhibition of its transcriptional activity without affecting p73 stability	[52]
WWP2	p73α and p73β	Oligomerization domain	p73 ubiquitination and its proteasomal degradation in	[53]

**Table 2:** Role of kinases in p73 inhibition.

Kinase	TAp73 Isoform	p73 Interaction Site	p73 Phosphorylation Site	Biological Responses	Refs
Cyclin A/CDK2, cyclin B/CDK2	p73α, p73β, p73γ	Two potential CRM sites (position 149, KKL; position 515, RAL)	Thr^86^	A decrease in the p73 transcriptional activity on the expression of the *p21^WAF1^* gene	[96]
PKA-Cβ	p73α	The C-terminal (469–636) and the N-terminal (63–130) regions	N-terminal region (residues 1–130)	A decrease in p73α transcriptional activity on *p21^WAF1^* and *Bax* genes	[97]
Plk1	p73α	NH2-terminal region	Thr^27^	A decrease in p73α transcriptional activity on *p21^WAF1^*, *Bax* and *MDM* genes and its pro-apoptotic activity	[98]
Plk3	p73α	NH2-terminal region (the amino acids 63-113)	AA residue(s) (63–113)	A decrease in p73α transcriptional activity on *p21^WAF1^* and the pro-apoptotic *Bax* and *p53AIP1* genes	[99]
Plk2	-	TA domain	Ser^48^	A decrease in p73 transcriptional activity	[100]
Aurora Kinase-A	-	DNA-binding domain	Ser^235^	A decrease in the ability of p73 for DNA-binding and its transactivation activity and inhibition of apoptosis	[101]
CK2	-	N-terminal domain	T^27^	A decrease in p73 transcriptional activity and an increase in the expression of several markers of cancer stem cells including Nanog, Oct4 and Sox2	[102]

**Table 3:** Drugs targeting post-translational modifications of p73.

Drug	FDA-Approved Drug	Target Pathway	Suggested Mechanism(s) of Action	Biological Responses	Refs
Protoporphyrin IX	Metvix^®^	Ubiquitination	Disruption p73/MDM2 and p73/MDMX complexes and inhibition of p73/Itch interaction [67].	Activation and stabilization of p73 and induction of p73-dependent apoptosis [134]. Upregulation of *NOXA* and *PUMA* expression [67].	[67,134,135]
Panobinostat	Farydac^®^	Ubiquitination	Decrease in the levels of Itch through E2F1 and myc-regulated transcription induction in the levels of miR106b	Increase in the expression of p73 protein and its downstream pro-apoptotic target *PUMA* with subsequent induction of apoptosis, without affecting the levels of p53.	[42]
Thymoquinone		Ubiquitination [62] Acetylation [136]	Decrease in the expression levels of various E3 ubiquitin ligases for p73 including Itch, Mdm2, FBXO45, and TRIM32 [62]. Decrease in the expression of SIRT1 protein and an increase in the expression of p300 [136].	Increase in the expression of p73 protein and induction of apoptosis.	[62,136]
Cisplatin	Platinol^®^	Phosphorylation	Binding of the SH2 domain of c-Abl to p73 protein, leading to its phosphorylation mainly on Tyr-99, Tyr-121 and Tyr-240 [137]. Activation of p38 [138]. Activation of c-Jun N-terminal kinase (JNK), which forms a complex with p73α and phosphorylates it at several serine/threonine residues, located within both the N- and C-terminal fragments [139].	Increase in the expression of p73 protein and the induction of apoptosis.	[137,138,139,140]
Doxorubicin	Doxil^®^	Phosphorylation [139] Acetylation [119]	The activation of JNK [139]. Acetylation of p73α by p300 predominately at lysine residues 321, 327, and 331 and this process requires the activation of tyrosine kinase c-Abl [119]. p300-mediated acetylation of p73 is also promoted by the binding of YAP1 to p73/p300 complex [141].	Increase in the expression of p73 protein [139] and activation of its pro-apoptotic activity [116,119].	[116,119,139,141]
Camptothecin	-	Phosphorylation	The activation of JNK [139].	Increase in the expression of p73 protein.	[139]
Arsenic trioxide	Trisenox^®^	Acetylation and phosphorylation [142]	Increase in p300–p73 interaction and p73α tyrosine phosphorylation.	Increase in the expression of p73α protein and a decrease in ΔNp73 activation of p73α pro-apoptotic activity.	[142]

## Data Availability

No new data were created or analyzed in this study. Data sharing is not applicable to this article.
